# Data verification of nationwide clinical quality registries

**DOI:** 10.1002/bjs5.50209

**Published:** 2019-08-19

**Authors:** L. R. van der Werf, S. C. Voeten, C. M. M. van Loe, E. G. Karthaus, M. W. J. M. Wouters, H. A. Prins

**Affiliations:** ^1^ Dutch Institute for Clinical Auditing Leiden the Netherlands; ^2^ Department of Surgery Leiden University Medical Centre Leiden the Netherlands; ^3^ Department of Surgery Erasmus University Medical Centre Rotterdam the Netherlands; ^4^ Department of Surgery Antoni van Leeuwenhoek Hospital Amsterdam the Netherlands

## Abstract

**Background:**

Clinical auditing is an emerging instrument for quality assessment and improvement. Moreover, clinical registries facilitate medical research as they provide ‘real world’ data. It is important that entered data are robust and reliable. The aim of this study was to describe the evolving procedure and results of data verification within the Dutch Institute for Clinical Auditing (DICA).

**Methods:**

Data verification performed on several (disease‐specific) clinical registries between 2013 and 2015 was evaluated. Sign‐up, sample size and process of verification were described. For each procedure, hospitals were visited by external data managers to verify registered data. Outcomes of data verification were completeness and accuracy. An assessment of the quality of data was given per registry, for each participating hospital. Using descriptive statistics, analyses were performed for different sections within the individual registries.

**Results:**

Seven of the 21 registries were verified, involving 174 visits to hospital departments. A step‐by‐step description of the data verification process was provided. Completeness of data in the registries varied from 97·2 to 99·4 per cent. Accuracy of data ranged from 88·2 to 100 per cent. Most discrepancies were observed for postoperative complications (0·7–7·5 per cent) and ASA classification (8·5–11·4 per cent). Data quality was assessed as ‘sufficient’ for 145 of the 174 hospital departments (83·3 per cent).

**Conclusion:**

Data verification revealed that the data entered in the observed DICA registries were complete and accurate.

## Graphical abstract

The aim of this study was to describe the procedure for data verification used by the Dutch Institute for Clinical Auditing (DICA), as well as the lessons derived from using the procedure. Between 2013 and 2015, seven of 21 audits were verified, and data were verified for completeness and accuracy.

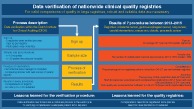

Data verification essential in clinical auditing

## Abstract (es)

**Antecedentes:**

La auditoría clínica es un instrumento emergente para la evaluación y mejora de la calidad. Además, los registros clínicos facilitan la investigación médica ya que proporcionan datos de la “vida real”. Es importante que los datos introducidos sean completos y fiables. El objetivo de este estudio fue describir la evolución y los resultados del procedimiento de verificación de datos en el seno del Instituto Holandés de Auditorias Clínicas (*Dutch Institute for Clinical Auditing*, DICA).

**Métodos:**

Se evaluó la verificación de datos realizada en varios registros clínicos (específicos de enfermedad) entre 2013‐2015. Se describió el sistema de registro, tamaño de la muestra y el proceso de verificación. Para cada procedimiento, los hospitales fueron visitados por administradores de datos externos para verificar los datos registrados. Los resultados de la verificación de datos fueron la integridad y la exactitud. Se proporcionó una evaluación de la calidad de los datos para cada registro en cada uno de los hospitales que participaron. Mediante la utilización de estadística descriptiva, se realizaron análisis de diferentes secciones para cada uno de los registros.

**Resultados:**

Siete de los 21 registros fueron verificados, lo que implicó un total 174 visitas a los departamentos de los hospitales. Se proporcionó una descripción paso a paso del proceso de verificación de los datos. La integridad de los datos en los registros varió entre 97,3‐99,4%. La exactitud de los datos varió entre 86,6‐97,0%. La mayoría de las discrepancias se observaron en las complicaciones postoperatorias (0,7‐7,5%) y clasificación ASA (7,5‐11%). La calidad de los datos se consideró “suficiente” en 145 de 174 (83%) departamentos hospitalarios.

**Conclusión:**

La verificación de los datos reveló que la introducción de datos en los registros DICA analizados era bastante completa y exacta.

## Introduction

Clinical auditing is predominantly an instrument for quality assessment and improvement in healthcare that can help to improve patient outcomes^1^, ^2^, ^3^, ^4^. Moreover, clinical registries facilitate evidence‐based medical research as they provide ‘real world’ data of patients. In 2009, the nationwide Dutch ColoRectal Audit (DCRA) was initiated by the Association of Surgeons of the Netherlands^5^. Together with the establishment of other clinical registries, this led to the foundation of the Dutch Institute for Clinical Auditing (DICA) in 2011^4^, ^5^, ^6^, ^7^. Today, 21 clinical registries are facilitated by DICA, and by 2016 more than 500 000 patients had already been registered^8^. The clinical registries are disease‐specific, and 16 of the 21 registries are surgical registries. In the Netherlands, all hospitals have an obligation to participate in these registries. Annually, a set of hospital‐specific outcomes are published on a public website, although only after approval by the board of each hospital^9^. These outcomes are used by policy‐makers, health insurance companies and patient federations to assess hospital performance.

A prerequisite for using these data for comparison of quality between hospitals is that the entered data are robust and reliable. The validity of entered data is essential, because they are used for medical and epidemiological outcome research. A recent validation study by Cundall‐Curry and colleagues^10^ emphasized the need for data uploaded to a national registry to be checked. Another validation of data quality in a national registry has been described by Linder *et al*.^11^, who showed that the database of the registry contained reliable data. A systematic approach for data verification in nationwide clinical registries has not been described. This study aimed to describe the procedure of data verification used by DICA, as well as the results of each procedure of data verification and the lessons learned from each procedure.

## Methods

This was a retrospective descriptive study of data verification in nationwide registries in the Netherlands, a high‐income country in western Europe with approximately 17 million inhabitants. Healthcare insurance is obligatory. Most secondary healthcare is provided in public hospitals. Secondary healthcare was provided in 71 hospitals in 2018. Since 2009, several nationwide registries have been set up by what is now known as DICA. In this study, data verifications performed between 2013 and 2015 were eligible.

### Data entry in the registries

Medical professionals have been responsible for the correct registration of their data in the registries. At the start of the DCRA, the majority of surgeons recorded the data themselves. Today, the recording of data is performed by medical specialists, trainees, physician assistants, data managers, research and administrative nurses. The medical specialist remains the final manager responsible for the quality of the data entered. Data are either uploaded in a web‐based system or delivered by the hospitals as a batch, at least once a year but preferably more often to facilitate quality improvements. Hospitals adhere to annual deadlines to deliver all data.

### Organizational structure of registries in DICA

Each registry is led by a clinical audit board, consisting of medical professionals mandated by their professional association. The registries also have a scientific committee, comprising representatives of the participating centres. Together with the scientific bureau of DICA, this scientific committee defines valid quality indicators, coordinates outcomes research, and is responsible for the quality of the data.

### Procedures to maintain the quality of registered data

In each clinical registry, the reliability of data is improved and verified in four ways. Verification systems are integrated in the web‐based survey, so that the registrar receives direct feedback on erroneous, missing or unlikely data items while entering the data.

DICA uses a signalling list that reports erroneous and missing data for all patients in a hospital. Clinical experts receive a weekly updated report with their outcomes for use in clinical auditing. This report also provides the number of registered patients and the completeness of the data, which can help to identify errors early. Finally, external data verification can contribute in determining the reliability of the data.

### External data verification

A first pilot project on external data verification was initiated by the Association of Surgeons of the Netherlands in 2014. This led to the formation of a data verification department at DICA that coordinates the procedures of external verification. An independent data verification committee was assigned, which consists of medical experts, a biostatistician, a deputy of the Dutch Health Care Inspectorate and a deputy from the Netherlands Patients Federation. Since the first procedure in 2014, the procedure of external data verification has been optimized based on experience gained during previous procedures.

External data verification is done by a third trusted party to guarantee the privacy of patients: Medical Research Data Management (MRDM), Deventer, the Netherlands. MRDM is NEN 7510:2011 and ISO 27001:2013 certified, and complies with privacy regulations in the Netherlands^12^.

### Pilot verification project

In the pilot project, the longest existing registries of DICA, the DCRA and the Dutch Upper Gastrointestinal Cancer Audit, were verified. In these verifications, 18 and 20 variables respectively were verified for all hospitals that participated in the registry. Per hospital, data for 20 patients were verified. With the experiences from the pilot project, the data verification procedure has been modified and was continued for other registries.

### Regular data verifications

#### *Patient and variable selection for verification*

The scientific committee sets selection criteria for the types of patient that should be included in the data verification, and selects the variables to be verified.

#### *Sign‐up*

Data verification was performed for each registry individually. All hospitals participating in the registry received an e‐mail invitation to participate. In the invitation letter the procedure, practical requirements and privacy of data verification were explained. Participation in data verification was voluntary and free of costs for the hospitals, although results were reported to the National Health Care Institute (Zorginstituut Nederland), which is responsible for public transparency of hospital‐specific quality information in the Netherlands.

#### *Sample sizes*

As previous studies were lacking, sample sizes were set arbitrarily in a consensus meeting with the data verification committee, which included a biostatistician. The preferred number of hospitals to verify for each registry was set at 15. The number of patients to verify in each hospital was based on a percentage of the annual hospital volume or a set number of patients, with a minimum of 30 patients.

#### *The process*

The process of data verification in hospitals was done manually by trained employees. They were all trained by DICA in both the verification procedure and the medical content. For each hospital, the completeness of the registration was evaluated, and the accuracy of data assessed.

#### *Completeness of the registry*

For the verification, the data set of a complete registration year was used. This data set was used for clinical auditing, to calculate the quality indicators for each hospital. To verify the completeness of the registry, hospitals were asked to provide a patient list derived from their administrative system. A sample of the list was compared with patients registered in the registry. Patients who were on the patient list but missing from the registry were registered as ‘absent’.

Different types of patient list were used. In the first verified registries, a patient list derived from the nationwide network and registry of histopathology and cytopathology in the Netherlands (PALGA network^13^) or a patient list with specific diagnosis–treatment combination (DBC) codes, as recorded by the hospital administration and insurance companies, was used. These DBC codes are used in the Netherlands for reimbursement of all costs of delivered care and are comparable with ICD codes.

Not all methods mentioned above proved to be applicable for every hospital because the PALGA system was not used in all hospitals and in some cases the DBC codes could differ between hospitals. Therefore it was decided that, for the studied verifications, hospitals could choose the type of patient list that fitted the aim of data verification and matched their system.

#### *Accuracy of the data*

To assess the accuracy of the data, the original data derived from the electronic patient records were compared with the data in the registry. For the hospitals, it was not possible to revise these data before data verification.

To register the accuracy of data, a web‐based survey was used in which the selected items to be verified were prefilled, based on the registered data. Each variable was assessed as ‘not discrepant’ or ‘discrepant’; missing data were assessed as ‘discrepant’. When discrepancies were observed, the correct information from the source data and an additional explanation of discrepancy had to be noted.

As a minimum, the variables needed to calculate two of the quality indicators were verified in all registries, including ‘the percentage of patients with severe complications’ and ‘the percentage of patients who died within 30 days after surgery’. For ‘severe complications’, different definitions were used among registries. Mostly, the definition was ‘complications leading to a prolonged hospital stay, a reintervention or death’. Another reason to verify a variable was the use of a variable in the case‐mix correction of outcome indicators, the ASA score, which is a scale of the preoperative fitness of patients^5^.

### Analysis of the data verification and results

In the process of analysing the data, an assessment of the observed discrepancies was done by an independent data manager and a medical researcher from DICA. Data for different hospitals were analysed separately. Completeness and accuracy of the data were assessed with descriptive statistics for different sections within the registries. Analyses were performed using IBM SPSS® version 23.0 (IBM, Armonk, New York, USA).

After evaluation of the discrepancies for each hospital by the data manager and medical researcher, the results of this evaluation were reported to the hospitals. In an adversarial process, it was possible for each hospital to give a response to the detected discrepancies. The independent verification committee had the final say.

A composite measure was defined for the conclusion of ‘sufficient quality’ or ‘insufficient quality’. *Table* 1 shows the criteria for the conclusion of ‘insufficient quality’ for one of the procedures. For some other procedures, small adjustments in thresholds were made due to a low number of patients or events.

**Table 1:** Factors leading to the label ‘insufficient quality’

Factor	Description
Completeness	Of all patients who met the inclusion criteria, more than 2 per cent (at least 2 patients) were not registered
Mortality	Of all patients who met the inclusion criteria, one or more patients died but were not registered at all or were not registered as ‘death’
Complication	Of all patients who had a complication, the complication was not registered in more than 5 per cent (at least 3 patients)
Reintervention	Of all patients who had a reintervention, the reintervention was not registered in more than 5 per cent (at least 3 patients)
Readmission	Of all patients who had a readmission, the readmission was not registered in more than 5 per cent (at least 3 patients)

The conclusion regarding the quality of the data and an anonymous summary report were communicated to the hospitals, to help them learn from the discrepancies and optimize their registration procedure. The results were also reported to the National Health Care Institute.

## Results

Since 2014, seven of the 21 registries have been verified individually. Information about the different verifications is shown in *Tables* 2 and 3.

**Table 2:** Characteristics and results of pilot verifications in 2013

	DCRA pilot	DUCA pilot
**Registry year of verification**	2013	2013
**Validation**		
Variables verified	20	18
Hospitals that signed up†	77 (88)	28 (88)
Hospitals verified	77	28
Patients verified per hospital	20	20
**Completeness**		
Missing patients†	271 of 9679 (2·8)	10 of 1251 (0·8)
Missed deaths	24	1
Missed patients with severe complications	55	2
**Accuracy**		
Total no. of patients in sample	1570	560
Discrepant deaths	5 (0·3)	0 (0)
Discrepant complications	117 (7·5)	17 (3·0)
Discrepant reinterventions	29 (1·8)	9 (1·6)
Discrepant ASA score	134 (8·5)	64 (11·4)
Discrepant radicality	4 of 415 (1·0)	11 of 235 (4·7)
**Objections**		
No. of hospitals	22	16

Values in parentheses are percentages.

^*^Sign‐up for the Dutch ColoRectal Audit (DCRA) and the Dutch Upper Gastrointestinal Cancer Audit (DUCA) was done together.

† Verification of completeness for DCRA and DUCA was done for all registered patients.

**Table 3:** Characteristics and results of verifications in 2014 and 2015

	Dutch Lung Cancer Audit	Dutch Audit for Carotid Interventions	Dutch Surgical Aneurysm Audit	Dutch Audit for Treatment of Obesity	Dutch Pancreatic Cancer Audit
**Registry year of verification**	2014	2015	2015	2015	2015
**Validation**					
Variables verified	17	6	9	9	13
Hospitals that signed up	29 of 43 (67)	36 of 53 (68)	39 of 60 (65)	12 of 20 (60)	12 of 19 (63)
Hospitals verified	15	13	14	12	12
**Completeness**					
Patients verified per hospital	± 26	± 22	± 21	± 35	± 30
Missing patients*	5 of 830 (0·6)	2 of 286 (0·7)	5 of 294 (1·7)	5 of 417 (1·2)	2 of 333 (0·6)
Missed deaths	0	0	0	0	0
Missed patients with severe complications	3	1	2	1	1
**Accuracy**					
Total no. of patients in sample	388	281	298	420	358
Discrepant deaths	0 (0)	2 (0·7)	0 (0)	0 (0)	0
Discrepant complications		13 (4·6)	22 (7·4)		
Discrepant severe complications	216 of 6596 (3·3)†			3 (0·7)	18 (5·0)
Discrepant reinterventions		0 (0)	0 (0)	3 (0·7)	
Discrepant readmissions			6 (2·0)		
**Objections**					
No. of hospitals	6		7		

Values in parentheses are percentages. Some cells are empty because the information was not available.

* Verification of completeness for these registries was done for all patients in the sample.

† Percentage calculated as the proportion of discrepant registrations of the total complications that could be registered for patients in the sample.

### Pilot verification project

In the pilot procedure, for all hospitals that signed up (77 in DCRA and 28 in DUCA) 18–20 variables and all patients eligible in 2013 were verified. This procedure was found to be very time‐consuming, logistically challenging and financially unfavourable. Therefore, for subsequent verifications a more limited set of variables was used. To limit the number of hospitals, 15 hospitals per registry was set; these hospitals were selected randomly by the third trusted party, MRDM.

### Regular verification project

#### *Patient and variable selection for verification*

The verified variables that were chosen differed between registries; all verified variables are shown in *Tables* 2 and 3.

#### *Sign‐up*

In the included seven data verification procedures, the percentage of hospitals signed up for verification varied between registries from 60 to 88 per cent.

In two verifications, some hospitals withdrew their sign‐up after selection because they were not able to comply with the conditions for verification (no time and priority for preparation). In two other verifications, fewer than 15 hospitals signed up. In 2015, an online survey was undertaken to investigate the reasons for refraining from signing up. The commonest reasons included that centres would have signed up but had forgotten, were too late or miscommunicated (8 of 21 answers), lack of time (4 of 21), and disagreed or did not comply with the legality of the procedure of verification (4 of 21).

#### *Sample size*

The number of patient records that were verified varied per registry, from 281 to 1570 (median 388).

#### *Completeness of the registry*

The percentage of unregistered patients varied from 0·6 to 2·8 per cent between registries. Details of these ‘missing patients’ are shown in *Tables* 2 and 3.

#### *Accuracy of the data*

Most discrepancies were observed in postoperative complications and ASA score (*Tables* 2 and 3). In 3·0–7·5 per cent of the total number of patients in the sample, registration of postoperative complications was discrepant, either wrongly registered or not registered. In 8·5–11·4 per cent of the total number of patients, an incorrect ASA score was registered or missing.

#### *Results of the procedures*

In 29 of 174 data verification processes performed, the quality of data was assessed as ‘insufficient’ according to the criteria. The number of hospitals that responded to the results or lodged an objection ranged from 6 to 22 per registry (*Tables* 2 and 3).

### Lessons learned from the results of each verification

An overview of the derived lessons is shown in *Table* 4. As concluded from discussions with the registrars, the most common discrepancies in the verifications seemed to be caused by unclear definitions and descriptions of variables. This was seen in six of seven verifications. The variables with the most discrepancies included the M status of the tumour, ASA score, the urgency of surgery, intraoperative complications, postoperative complications, reinterventions, and the number of days in the ICU. Incorrect inclusion and incorrect exclusion of patients in the registries were also observed.

**Table 4:** Lessons learned from the verifications

	Dutch Surgical Colorectal Audit (pilot)	Dutch Upper Gastrointestinal Cancer Audit (pilot)	Dutch Lung Cancer Audit	Dutch Audit for Carotid Interventions	Dutch Surgical Aneurysm Audit	Dutch Audit for Treatment of Obesity	Dutch Pancreatic Cancer Audit
**Lessons derived for the procedure of data verification**							
More extensive training for verification employees needed							x
Patient list not suitable			x	x	x	x	x
Selection of hospitals: too many hospitals verified	x	x					
Selection of variables: too many variables verified	x	x					
Time‐consuming to evaluate completeness for all patients rather than a sample			x				
Selection of patients: too few patients verified				x	x	x	
Privacy of patient records during the procedure was complex	x	x					
Criteria for ‘sufficient/insufficient’ need to be set before start of data verification	x	x					
Criteria for ‘sufficient/insufficient’ need to be changed	x	x	x				
Criteria for ‘sufficient/insufficient’ are without nuance			x	x	x	x	x
Data verification has to become a continuous process in the audit cycle	x	x	x	x	x	x	x
**Lessons derived for registrars**							
Need to fill in all variables, also when not required			x				
Complications need to be registered more precisely			x	x	x	x	x
ASA score needs to be registered as described in the anaesthesia report	x	x					
Date of surgery has to be registered more precisely				x		x	
Date of discharge has to be registered more precisely							x
Hospitals must adhere to inclusion and exclusion criteria					x		
**Lessons derived for the audits**						x	
Need for clear definitions of variables	x	x	x	x	x		x
Error in data structure discovered						x	

## Discussion

This study showed that verification of the completeness and accuracy of the registry is essential. The strength of the described process is that a dedicated team within the audit organization initiates and coordinates nationwide data verifications of the registries. By learning from every verification, the process of verification was improved continuously. Data verification may help to improve the survey of the registries and thereby contribute to higher quality data sets. The most important lesson derived from the verification is the need for clear definitions of variables.

In the first verification procedures, many of the missing patients had severe complications or had died. These discrepancies may have happened because hospitals were afraid to be criticized if they registered all patients with complications. Another explanation might be that hospitals were not capable of following some of their patients with complications, as these patients are often treated on different wards (such as the ICU) or even transferred to another hospital. Because the registry is used to compare hospitals, it is imperative that all hospitals have a complete registry. Verification of data completeness may stimulate hospitals to adhere to the proposed rules of data entry.

The verification of data accuracy is also important. One of the requirements for accurate data is the use of clear definitions for multi‐interpretable variables. Many discrepancies, however, were seen for simple, uni‐interpretable variables, such as date of surgery and date of discharge. Because length of stay and waiting times are frequently used as quality indicators, these results indicate that simple variables should also be verified.

By detecting common discrepancies, such as those resulting from unclear description of items, the survey could be improved by the clarification of definitions, to prevent incorrect data in the future. Furthermore, by reporting erroneous data, registrars in hospitals can learn lessons and improve the registrations. A side‐effect of integrated data verification in the cycle of clinical auditing might be that it stimulates hospitals to register correctly, because they know their data will be verified. This so‐called Hawthorne effect describes improved results that might result from increased awareness for an outcome, in this situation the collection of correct data^14^.

All of these mechanisms could benefit the quality of the data sets and may lead to more valid registries and more reliable data for outcome research. Valid registries are important because the results of quality indicators are publicly available for policy‐makers, health insurance companies and patient federations.

The described process also has limitations, which could be improved upon. Hospitals that might intentionally register incorrectly or incompletely were not identified by the present procedure because signing up for data verification was voluntary. Hospitals can influence their published results by intentionally registering incorrect or incomplete data. This might be a problem because the results are used for clinical auditing and comparisons between hospitals. A counter‐argument for making verification mandatory is that some medical specialists already feel criticized by clinical auditing as it takes some time. Forcing them to have data verification may create resistance in the field. For the integrity of verification, however, it is desirable that the National Health Care Institute (Zorginstituut Nederland) declares the process of data verification mandatory. Another possibility could be that details on sign‐up and participation in data verification become publicly transparent, and could be used to assess the validity of indicator results for individual hospitals.

Another limitation in the present procedure is the struggle to verify the completeness of the registry. At present, hospitals are free to choose which patient list they provide. A frequently used patient list is one extracted from the electronic patient record system. This strategy is not protected against flaws, because this list could be the same as that used to select patients for registration. A further disadvantage of this system is that hospitals could manipulate the patient list if they wanted to ‘hide’ patients with severe complications. The results of the verifications, however, showed that use of these self‐provided lists succeeded in identifying unregistered patients.

To improve registries further and provide valuable, verified, benchmark data to all parties involved, DICA aims to develop a system in which data verification becomes a continuous process, as part of the registry. For this purpose, data verification is included in the annual budget. This year will be the first in which data verification will be repeated in two registries that have been verified previously, 3 years ago.

Regarding the optimal sample size for verification, difficulties in finding a balance between the cost aspect and certainty of the verification were experienced in the past. In the near future, a pilot will be started to verify clinical outcome registry data in a more automated process. This pilot aims to select patients with high risk of discrepancy^15^. The hypothesis is that verification of these high‐risk patients will lead to a higher sensitivity for discrepancies when the same sample size is used as in the present procedure. As sample size directly influences costs, this procedure will be more cost‐effective. This pilot is to be funded by Stichting Kwaliteitsgelden Medisch Specialisten (SKMS), a Dutch foundation with a policy of improving quality for medical specialists, and which is part of the Dutch Federation of Medical Specialists.

For most verifications, the absence of clear and uniform definitions of items led to the most discrepancies. DICA will make an important improvement by creating uniform, clear and correct definitions for items in all registries. Recently, a project was launched for this purpose. In this project, as many items as possible will be defined equally in all registries, with an attempt to use existing guidelines, classifications and definitions, such as the definitions used in SNOMED Clinical Terms and ICD‐10 codes. SKMS also supports this project.

It is expected that registration of data will become increasingly automated in the near future. The authors envisage that correct data from electronic patient records will be uploaded automatically to the registry without the use of data managers.

## Collaborators

R. Veenstra (Medical Research Data Management, Deventer, the Netherlands) is a collaborating author.
